# Cold-acclimation limits low temperature induced photoinhibition by promoting a higher photochemical quantum yield and a more effective PSII restoration in darkness in the Antarctic rather than the Andean ecotype of *Colobanthus quitensis* Kunt Bartl (Cariophyllaceae)

**DOI:** 10.1186/1471-2229-12-114

**Published:** 2012-07-24

**Authors:** Luisa Bascuñán-Godoy, Carolina Sanhueza, Marely Cuba, Gustavo E Zuñiga, Luis J Corcuera, León A Bravo

**Affiliations:** 1 Laboratorio de Fisiología Vegetal, Centro de Estudios Avanzado Zonas Áridas, La Serena, Chile; 2 Departamento de Botánica, Facultad de Ciencias Naturales y Oceanográficas, Universidad de Concepción, Casilla 160-C, Concepción, Chile; 3 Laboratorio de Biotecnología y Estudios Ambientales, Departamento de Ciencias y Tecnología Vegetal, Escuela de Ciencias y Tecnología, Universidad de Concepción, Campus los Ángeles, Casilla 160-C, Los Ángeles, Chile; 4 Laboratorio de Fisiología Vegetal, Facultad de Química y Biología, Universidad de Santiago de Chile, Casilla 307 Correo 2, Santiago, Chile; 5 Laboratorio de Fisiología y Biología Molecular Vegetal, Instituto de Agroindustria, Departamento de Ciencias Agronómicas y Recursos Naturales, Facultad de Ciencias Agropecuarias y Forestales and Center of Plant, Soil Interaction and Natural Resources Biotechnology, Scientific and Technological Bioresource Nucleus, Universidad de La Frontera, Casilla 54D, Temuco, Chile

**Keywords:** Antarctic plants, Andean plants, Cold-induced-photoinhibition, Recovery, PSII restoration, D1 cycle, Photoprotection

## Abstract

**Background:**

Ecotypes of *Colobanthus quitensis* Kunt Bartl (Cariophyllaceae) from Andes Mountains and Maritime Antarctic grow under contrasting photoinhibitory conditions, reaching differential cold tolerance upon cold acclimation. Photoinhibition depends on the extent of photodamage and recovery capability. We propose that cold acclimation increases resistance to low-temperature-induced photoinhibition, limiting photodamage and promoting recovery under cold. Therefore, the Antarctic ecotype (cold hardiest) should be less photoinhibited and have better recovery from low-temperature-induced photoinhibition than the Andean ecotype. Both ecotypes were exposed to cold induced photoinhibitory treatment (PhT). Photoinhibition and recovery of photosystem II (PSII) was followed by fluorescence, CO_2_ exchange, and immunoblotting analyses.

**Results:**

The same reduction (25%) in maximum PSII efficiency (Fv/Fm) was observed in both cold-acclimated (CA) and non-acclimated (NA) plants under PhT. A full recovery was observed in CA plants of both ecotypes under dark conditions, but CA Antarctic plants recover faster than the Andean ecotype.

Under PhT, CA plants maintain their quantum yield of PSII, while NA plants reduced it strongly (50% and 73% for Andean and Antarctic plants respectively). Cold acclimation induced the maintenance of PsaA and Cyt *b6/f* and reduced a 41% the excitation pressure in Antarctic plants, exhibiting the lowest level under PhT. xCold acclimation decreased significantly NPQs in both ecotypes, and reduced chlorophylls and D1 degradation in Andean plants under PhT.

NA and CA plants were able to fully restore their normal photosynthesis, while CA Antarctic plants reached 50% higher photosynthetic rates after recovery, which was associated to electron fluxes maintenance under photoinhibitory conditions.

**Conclusions:**

Cold acclimation has a greater importance on the recovery process than on limiting photodamage. Cold acclimation determined the kinetic and extent of recovery process under darkness in both *C. quitensis* ecotypes. The greater recovery of PSII at low temperature in the Antarctic ecotype was related with its ability to maintain PsaA, Cyt *b6/f* and D1 protein after photoinhibitory conditions. This is probably due to either a higher stability of these polypeptides or to the maintenance of their turnover upon cold acclimation. In both cases, it is associated to the maintenance of electron drainage from the intersystem pool, which maintains Q_A_ more oxidized and may allow the synthesis of ATP and NADPH necessaries for the regeneration of ribulose 1,5-bisphosphate in the Calvin Cycle. This could be a key factor for *C. quitensis* success under the harsh conditions and the short growing period in the Maritime Antarctic.

## Background

The balance between absorbed and photochemically converted energy for metabolism is critical in photosynthetic organisms [1]. The absorption of excessive energy with respect to energy used in photosynthesis may induce photoinhibition [2]. Photoinhibition is a consequence of either reversible down-regulation of PSII through the dissipation of excess absorbed energy or the irreversible inactivation of PSII and damage to the D1 reaction center protein [2,3]. However, D1 is also degraded under non-photoinhibitory conditions and it is continuously replaced by newly synthesized protein. When plants are exposed to excessive high light conditions or when new protein synthesis is impaired by unfavorable environmental stress conditions, the inactivation rate exceeds the capacity for its repair. Under this condition, the content of functional D1 protein is depleted, resulting in photodamage of PSII [4,5]. The actual extent of photoinhibition “*in vivo”* depends on the balance between inactivation of D1 and the repair process, which involves insertion of new D1 molecules into the thylakoid and their incorporation into the PSII complex [6]. Light is a requirement for PSII function restoration. Recovery from photoinhibition does not occur in darkness, mainly due to impaired thylakoid protein synthesis [7].

In addition, recovery from photoinhibition is strongly temperature dependent [8-10]. Under low temperature, the inhibition of D1 repair process has been postulated as the principal mechanism of photoinhibition, because “*de novo”* D1 synthesis is impaired [11]. Cold acclimation results in an increase in photosynthesis capacity at suboptimal temperature [2]. Previous studies have demonstrated that increased tolerance to photoinhibition in cereals is a result of growth and development under conditions that induce a high PSII excitation pressure, which reflects the redox poise of the intersystem electron transport chain [12-14]. This is supported by earlier studies that have reported correlations between tolerance to photoinhibition and the redox state of Q_A,_ the first stable quinone electron acceptor of PSII [15,16]. Recovery in the photosynthesis capacity is closely related with the activation of electron sink process, which can induce a higher relative oxidized state of Q_A_, reducing ROS (reactive oxygen species) induction probability [17,18].

Thermal dissipation of excess absorbed energy, at antenna level, is a fast and efficient protective strategy which prevents over-reduction of Q_A_[19]. Thermal dissipation is measured through non-photochemical quenching (NPQ) [20]. NPQ has been shown to be composed of at least two components with different relaxing time scales. The fast relaxing component NPQ_f_, also called qE, is rapidly relaxed after dark. It requires thylakoid lumen acidification, zeaxanthin synthesis in the xanthophylls cycle and protonation PsbS protein [21-23]. Under prolonged light stress, qE is replaced by a sustained, slowly reversible component NPQ_s_ or photoinhibitory component (qI). This component is less characterized and it has been linked to retention of zeaxanthin under dark and photodamage of D1 [19]. The relaxation of NPQ and the epoxidation of zeaxanthin are important to the first phase of the recovery process [3,24,25].

*Colobanthus quitensis* (Kunth) Bartl. Caryophyllaceae is one of only two angiosperm species to have naturally overcome the geographical and environmental impediments for colonization of the Antarctic [26]. *C. quitensis* extends from the Maritime Antarctic and along the Andes Mountains to Ecuador, with one site in Mexico [27]. In the Antarctic, *C. quitensis* grows as a perennial herb which develops its vegetative and reproductive cycle typically between December and March, with frequent average daily air temperatures usually between 0°C and 6°C, and the minima are between −2°C and −4°C [28,29]. The photoperiod reaches about 21/3 light/dark hours in December in the Maritime Antarctic, with a vast majority of cloudy days (usually 300 to 600 μmol photons m^-2^ s^-1^). Clear days with much higher photosynthetic photon flux density (PPFD) reach about 20% of summer days [26,30,31]. *C. quitensis* usually grows above 2500 m a.s.l. in the Andes Mountains [32]. In the Andes of Central Chile, its life cycle is developed from October to April (during the snow-free period) with shorter photoperiods than in the Antarctic. This ecotype grows under wider diurnal temperature oscillations (usually between 0-22°C) and frequently exposed to high PPFD at noon (about 2000 μmol photons m^-2^ s^-1^) [33,34].

It has been recently proposed that differences in morphological and leaf attributes due to acclimation to each particular environment rely on different photoprotective mechanisms [35]. Molecular studies of the ITS (Internal Transcriber Spacers) of both populations of *C. quitensis* demonstrated high ITS similarity among both accessions. Based on morphological and physiological differentiation under common garden experiments, they are now considered ecotypes [36]. Upon 21 days at 4°C, the Antarctic ecotype exhibited higher cold resistance capacity than the Andean one, reaching a LT_50_ (lethal temperature at which 50% of plants died) 4.5°C lower than the Andean ecotype [36]. Previous studies have reported that both ecotypes have similar optimal temperature for photosynthesis ranging from 17 to 24°C depending on growing conditions [30,37] However, low temperature exposure (cold acclimation) improves the Antarctic ecotype responses to photoinhibitory conditions, such as a combination of high light intensity and low temperature [38].

Most of the above studies on *C. quitensis* have considered short term exposure to photoinhibitory conditions. However, the extent of damage, the recovery phase, and how low temperature and dark may limit these processes have not been studied. Therefore, the main objective of this work is to understand how cold acclimation affects the extent of photoinhibitory damage and recovery in two ecotypes of *C. quitensis.* We hypothesized that cold acclimation increases resistance to low-temperature-induced photoinhibition by limiting photodamage and/or promoting recovery under cold, especially in the cold hardiest ecotype.

## Results

### Cold acclimation effect on recovery kinetics of maximum PSII efficiency (Fv/Fm) from a cold-induced photoinhibitory treatment (PhT)

**Figure 1:**
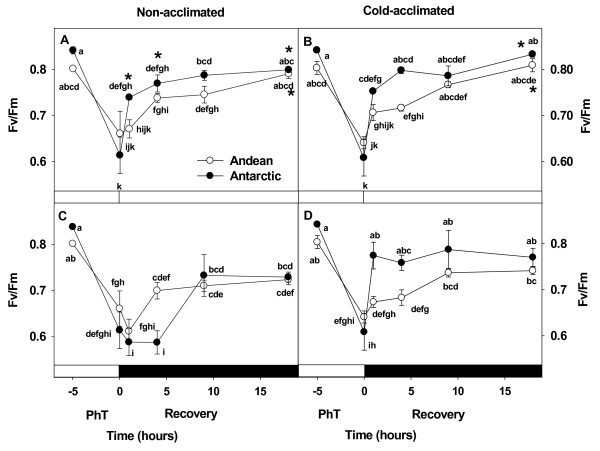
** Effect of cold acclimation on recovery kinetics of Fv/Fm under light (A,B) and dark (C,D) from PhT in plants of both ecotypes of*C. quitensis*.** Recovery was monitored after 1, 4, 9, and 18 hours at 4°C. Mean values ± SE were calculated from five independent measurements of different individual. Different letters represent significant differences between growing conditions, ecotypes and treatment *P<*0.05 using three-way ANOVA. * indicated significant differences between low light (50 μmol photons m^-2^ s^-1^) and dark recovery, for each ecotype and growth temperature condition, *P<*0.05 performed by one-way ANOVA.

Fv/Fm variation upon PhT and recovery depended on ecotype, acclimation temperature, and treatment (significant interaction *P* <0.05). After 5 hours of exposure to 4°C under high light, all plants experienced similar substantial photoinhibition, with a 25% decline in Fv/Fm (Figure 1).

Fv/Fm recovery from PhT depended on the light environment. Recovery under low light (50 μmol photons m^-2^ s^-1^) allowed a complete and faster Fv/Fm restoration in the NA Antarctic ecotype with respect to recovery under dark (Figure 1A, C). Significant differences in Fv/Fm values between low light and dark recovery of NA plants were obtained at 1, 4, and 18 hours of recovery in the Antarctic ecotype. However, the Andean ecotype only showed differences at 18 h of recovery. In both ecotypes of NA plants, restoration of PSII function was not complete at 18 h of recovery under darkness.

Cold acclimation determined full Fv/Fm restoration under darkness in both ecotypes. Cold acclimation also increases the velocity of Fv/Fm restoration in the Antarctic ecotype. Total restoration of Fv/Fm in CA Antarctic plants occurred during the first hour of darkness, reaching about 90% of the initial Fv/Fm, without significant differences with the value reached under low light at the same time. In contrast, Fv/Fm of Andean plants increased linearly and slowly during the recovery period, reaching initial Fv/Fm values at about 9 hours of darkness, similar than under low light recovery.

### Effect of cold acclimation on photoinhibition and dark recovery of Chlorophyll fluorescence parameters

In order to further understand how cold acclimation affect recovery of photoinhibited PSII in *C. quitensis*, photochemical and non-photochemical fluorescence parameters were studied before PhT (Photoinhibitory treatment), immediately after PhT and at 12 h of recovery (chosen as full recovery because non-statistically significant differences were observed between 9 and 18 hours of dark recovery). The values of PhT and recovery were compared with those obtained from non-photoinhibited plants performed at 4°C (initials parameters).

**Figure 2:**
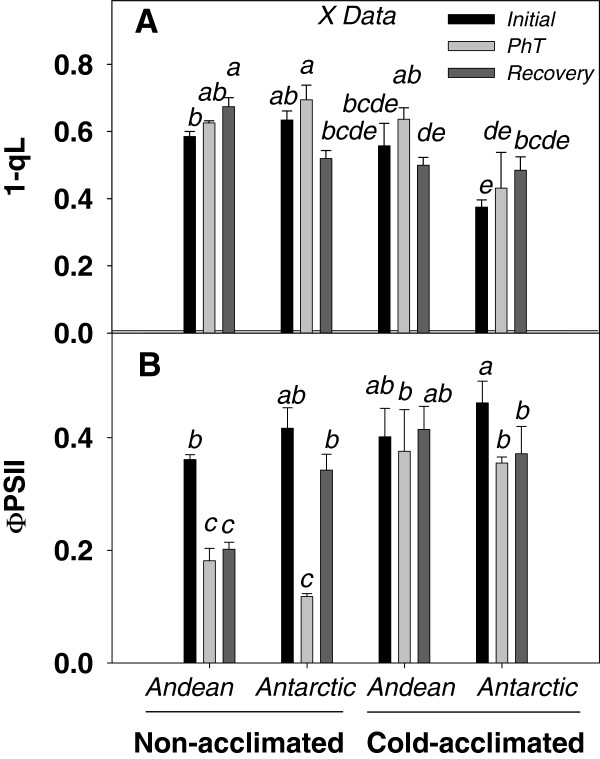
** Effect of cold acclimation on photochemical fluorescence parameters under PhT and dark recovery in plants of both ecotypes of*C. quitensis*.** 1-qL (**A**) is the proportion Q_A_ in the reduced state, and ΦPSII (**B**) is quantum yield of PSII. Measurements were done at 4°C using an actinic light of 900 μmoles of photons m^-2^ s^-1^. Results were calculated from five independent measurements of different plants. Bars show mean values ± SE. Different letters represent significant differences between growing conditions, ecotypes and treatment *P<*0.05 using three-way ANOVA.

Cold acclimation reduced a 41% the excitation pressure of PSII (1-qL) in the Antarctic ecotype (Figure 2A), while a slight tendency to increase 1-qL in photoinhibited plants was observed in NA plants of both ecotypes. Interestingly, CA Antarctic plants showed the lowest average of 1-qL under PhT. This was a 30% lower respect to NA and CA Andean plants (Figure 2A). During the recovery, NA Andean plants exhibited the highest excitation pressure, while 1-qL decreased to initial values in NA Antarctic and CA plants of both ecotypes.

Quantum yield of PSII (ΦPSII) responses under photoinhibitory treatments were dependent on acclimation temperature (*P* <0.05) and ecotype (*P* <0.05). However, the acclimation response was similar for both ecotypes (non-significant interaction between ecotype and acclimation temperature (*P* <0.05). ΦPSII was reduced a 50% in the Andean and a 73% in the Antarctic ecotype under PhT in NA state. In spite the stronger ΦPSII reduction of the Antarctic ecotype under PhT, it was able to recover its initial yield after dark recovery, while the Andean ecotype was not (Figure 2B).

**Figure 3:**
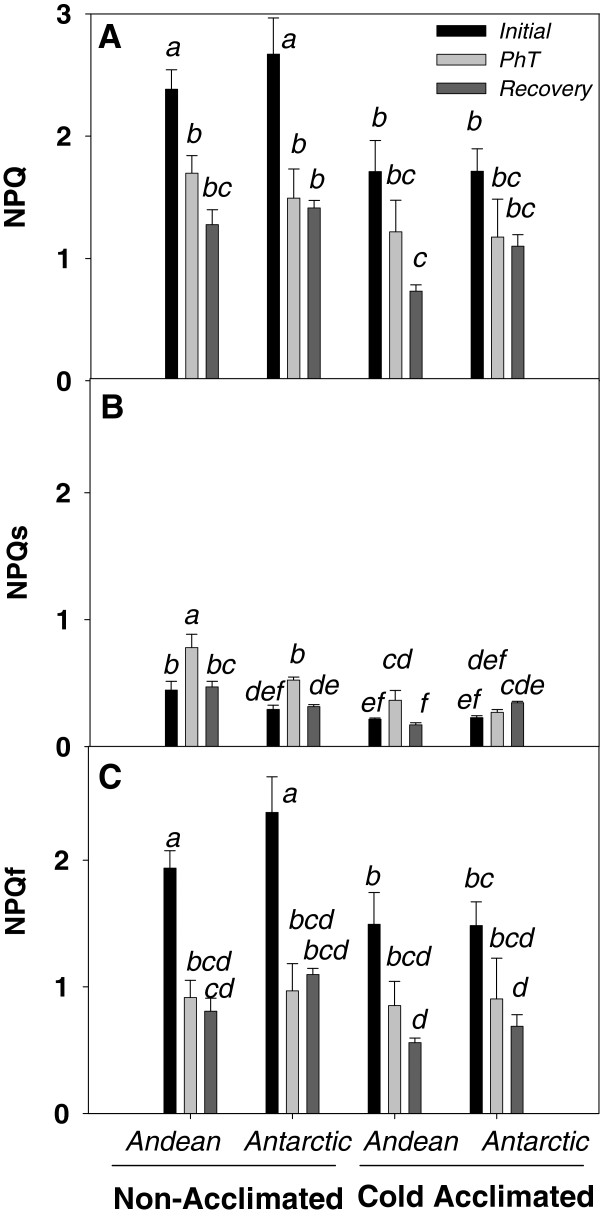
** Effect of cold acclimation on total non-photochemical quenching NPQ (A), slow relaxing component NPQ**_**s**_**(B) and fast relaxing component NPQ**_**f**_**(C) under PhT and after the recovery period in both ecotypes of*C. quitensis*.** Measurements were done using an actinic light of 900 μmoles of photons m^-2^ s^-1^ at 4°C. Bars show mean values ± SE. They were calculated from five independent measurements of different plants. Different letters represent significant differences between growing conditions, ecotypes and treatment *P<*0.05 using three-way ANOVA.

**Figure 4:**
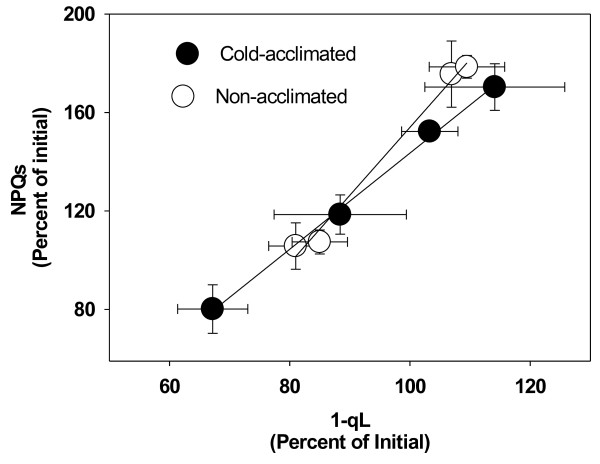
** Relationship between relative variation in the photoinhibitory component of NPQ (NPQs) and relative changes in excitation pressure (1-qL).** Values were calculated as percent of non-photoinhibited plants. Filled circles (●) correspond to CA plants and empty circles (○) to NA plants. Mean values ± SE were calculated from five independent measurements.

PhT did not induce a higher level of NPQ and NPQ_f_ (Figure 3A, C) with respect to values of non-photoinhibited plants. In contrast, PhT induced higher levels of NPQ_s_ in these plants. The effects of PhT on NPQ_s_ were dependent on temperature acclimation and ecotype (significant interaction *P* <0.05). Only CA Antarctic plants maintained similar NPQ_s_ values than control non-photoinhibited plants. The greatest NPQ_s_ was observed in NA plants of the Andean ecotype, where NPQ_s_ achieved similar values than NPQ_f_. After the recovery period, NPQ_s_ tended to decrease to the initial values in both NA and CA plants (Figure 3B). A similar strong correlation (r^2^ = 0.95) between the relative changes (respect to the initial value) in NPQ_s_ and 1-qL was observed in NA and CA plants under PhT and recovery (Figure 4). Therefore, it seems that cold acclimation does not decrease PSII susceptibility to chronic photoinhibition at a given variation of excitation pressure.

### Effect of cold acclimation on chlorophyll content and thylakoids proteins changes after photoinhibitory treatment and dark recovery

**Figure 5:**
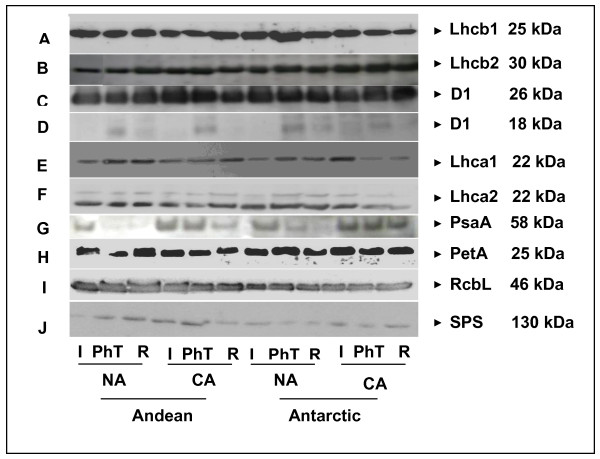
** Effect of cold acclimation on Lhcb1 (A), Lhcb2 (B), D1 (C), and its degradation product of 18 kDa (D), Lhca1(E), Lhca2 (F), PsaA (G), Cyt*b6/f*(H), Rubisco (I) and SPS (J) levels under PhT and after the recovery period in plants of both ecotypes of*C. quitensis*.** The figure shows the western-blot for every protein with its respective molecular weight. Densitometric analysis was performed to three independent westernblots.

There was a significant higher chlorophyll (Chl) *a* content (13%) in the Andean ecotype, while no significant difference between ecotypes was observed in Chl *b*, consequently, there is a higher average Chl *a/b* ratio in the Andean (3.9) than in the Antarctic (2.6) ecotype (Table 1). Chl *a* decreased about 30% in both CA ecotypes; while Chl *b* was reduced 35% upon cold acclimation only in the Antarctic ecotype. As a consequence, cold acclimation significantly reduced the Chl *a*/*b* ratio only in the Andean ecotype. After the PhT, the Antarctic ecotype tended to maintain its Chl levels (in both NA and CA state), while NA Andean plants exhibited large reductions in both Chl *a* and Chl *b*. CA Andean plants showed a significant reduction only in Chl *b.* Additionally, NA Andean plants were not able to reestablish their initial Chl levels during dark recovery. Higher levels of Lhcb1 and Lhcb2 from PSII antenna complex were observed in Antarctic plants (Figure 5A, B). D1 protein levels were similar in NA and CA stages of both ecotypes (Figure 5C). After PhT, the 18 kDa degradation product of D1 increased significantly (Figure 5D), but they were reestablished to the initial level after recovery. In NA Andean plants, the 18 kDa D1 degradation product increased concomitantly with a 30% decrease of D1 protein (Figure 5C, D).

**Table 1:** **Effect of cold acclimation on chlorophyll content of plants of both ecotypes of*C. quitensis*subjected to PhT and a recovery period**

			**Chl*a*(μg g-1 FW)**	**Chl*b*(μg g-1 FW)**	**Chl*a*/Chl*b***
**Non-acclimated**	**Andean**	**Initial**	767 ± 36(a)	208 ± 29(abc)	3.9 ± 0.7(abc)
		**PhT.**	490 ± 22(cd)	135 ± 15(d)	3.7 ± 0.4(abc)
		**Recovery**	527 ± 37(cd)	120 ± 16(d)	4.6 ± 0.9(a)
	**Antarctic**	**Initial**	660 ± 25(b)	258 ± 21(a)	2.6 ± 0.3(cd)
		**PhT.**	569 ± 25(c)	247 ± 29(a)	2.4 ± 0.4(d)
		**Recovery**	744 ± 26(ab)	260 ± 31(a)	2.9 ± 0.3(bcd)
**Cold-acclimated**	**Andean**	**Initial**	520 ± 23(cd)	242 ± 20(ab)	2.2 ± 0.3(d)
		**PhT.**	441 ± 23(de)	133 ± 12(d)	3.3 ± 0.2(bcd)
		**Recovery**	704 ± 46(ab)	236 ± 16(ab)	3.0 ± 0.0(bcd)
	**Antarctic**	**Initial**	473 ± 27(de)	166 ± 23(cd)	3.0 ± 0.6(bcd)
		**PhT.**	393 ± 34(d)	180 ± 21(bcd)	2.2 ± 0.2(d)
		**Recovery**	470 ± 36(de)	171 ± 19(cd)	2.8 ± 0.1(bcd)

Results show mean values ± SE of two separate experiments with 3 replicates each. Different letters represent significant differences between acclimation, treatment and ecotypes *P<*0.05 using three-way ANOVA.

Lhca1 and Lhca2 levels were maintained in response to PhT in NA and CA Andean plants. Cold acclimation induces a 40% of reduction in the amount of these two proteins from PSI antenna in Antarctic plants subjected to PhT. Normal level of these proteins were not reestablished after the recovery period (Figure 5E, F). In contrast, PsaA protein level was largely reduced (about 50%) in NA plants during PhT, and in NA plants and CA Andean plants during the dark recovery, while it was maintained constant in CA Antarctic plants (Figure 5G). Although, both ecotypes experienced 30% of increment on Cyt *b6/f* (PetA subunit) after CA, the highest level was observed in CA Antarctic plants (Figure 5H). In CA and NA Andean plants the Cyt *b6/f* level decreased about 50% and 20%, respectively during PhT. Antarctic plants on the other hand, tended to maintain Cyt *b6/f* levels independently of the treatment.

In order to associate the effects of PhT with carbon metabolism, the first enzyme of the Calvin cycle and the key enzyme of sucrose biosynthesis were immunologically studied. NA Andean plants exhibited the highest level of large subunit of Rubisco (ribulose bisphosphate carboxylase/oxigenase) (Figure 5I). Rubisco remained nearly constant after PhT, and increased a 25% during the recovery of both CA ecotypes. Cold acclimation increased Sucrose phosphate synthase (SPS) levels in the Andean ecotype, but not in Antarctic one. The densitometric analysis showed about 20% of increment of SPS observed after recovery in CA Antarctic plants (Figure 5J).

### Effect of photoinhibitoty treatment and recovery on net photosynthesis

**Figure 6:**
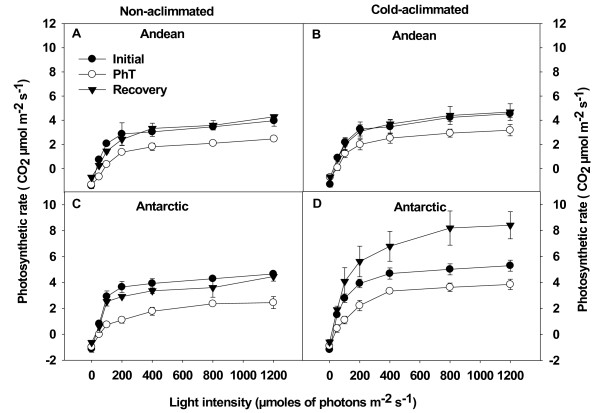
** Effect of cold acclimation on light response curves of net photosynthesis under PhT and after the recovery period.** NA (**A**, **C**) and CA (**B**, **D**) plants of both ecotypes of *C. quitensis* were subjected to PhT and dark recovery. Net photosynthesis was measured after each treatment and the respective non photoinhibited controls at 4°C under 360 ppm CO_2_ at different PPFDs. Results are mean values ± SE of two separate experiments with 3 replicates each.

Light response curves of net photosynthesis (A) at 4°C were studied before, immediately after PhT, and at the end of recovery period in CA and NA plants of both ecotypes (Figure 6). Cold acclimation did not induce significant differences in the maximal rate of net photosynthesis (A_max_) measured at 4°C in both ecotypes.

The reduction in A_max_ after PhT was lower in CA plants (28% for both ecotypes) than in NA plants (34% and 51% for Andean and Antarctic plants, respectively). After the recovery period, A_max_ was reestablished to initial values in NA and Andean CA plants, while CA Antarctic plants exhibited a greater level respect to initial values. Therefore, net photosynthesis recovery was strongly dependent on the ecotype and acclimation temperature (significant interaction *P* <0.05). CA increased the quantum yield of CO_2_ uptake (ΦCO_2_) by 47% in Andean and 90% in Antarctic plants, respectively (Table 2, Figure 6). PhT reduced significantly the ΦCO_2_ on both NA and CA plants. These values were totally recovered after 12 hours of darkness (Table 2, Figure 6). The PhT increased the light compensation point (LCP) in NA plants (*P* <0.05), while only slightly differences were observed in CA Andean plants. Despite this, values were reestablished after the recovery period on both NA and CA plants. Another interesting change was observed in dark respiration (Rd) after the recovery period. About a 45% reduction in Rd was observed in almost all groups of plants. Only in CA Antarctic plants Rd did not change significantly.

**Table 2:** **Effect of cold acclimation on photosynthetic parameters of plants of both ecotypes of*C. quitensis*subjected to PhT and a recovery period**

			**Photosynthetic parameters**			
	**Ecotype**	**Treatment**	**Rd**	**ΦCO**_**2**_	**A**_**max**_	**LCP**
**Acclimation**			**(μmol CO**_**2**_**m**^**-2**^**s**^**-1**^**)**	**(mol mol**^**-1**^**photons)**	**(μmol CO**_**2**_**m**^**-2**^**s**^**-1**^**)**	**(μmol photons m**^**-2**^**s**^**-1**^**)**
**Non- acclimated**	**Andean**	**Initial**	−1.4 ± 0.2 (a)	0.034 ± 0.004 (de)	3.9 ± 0.5 (cde)	31 ± 1 (bcd)
		**PhT.**	−1.3 ± 0.3 (a)	0.021 ± 0.002 (e)	2.5 ± 0.2 (f)	68 ± 1 (a)
		**Recovery**	−0.7 ± 0.1 (de)	0.025 ± 0.003 (de)	4.3 ± 0.2 (bcde)	29 ± 5 (bcde)
	**Antarctic**	**Initial**	−1.1 ± 0.2 (abcd)	0.040 ± 0.003 (cd)	4.7 ± 0.5 (bcd)	23 ± 3 (cdef)
		**PhT.**	−1.0 ± 0.1 (abcde)	0.020 ± 0.003 (e)	2.3 ± 0.3 (f)	42 ± 4 (b)
		**Recovery**	−0.6 ± 0.1 (e)	0.039 ± 0.002 (cd)	4.5 ± 0.4 (bcde)	20 ± 6 (def)
**Cold- acclimated**	**Andean**	**Initial**	−1.3 ± 0.2 (ab)	0.050 ± 0.010 (bc)	4.5 ± 0.5 (bc)	24 ± 4 (cdef)
		**PhT.**	−0.7 ± 0.1 (de)	0.025 ± 0.006 (de)	3.2 ± 0.5 (ef)	36 ± 10 (bc)
		**Recovery**	−0.7 ± 0.1 (e)	0.051 ± 0.005 (bc)	4.7 ± 0.7 (bcd)	21 ± 8 (def)
	**Antarctic**	**Initial**	−0.8 ± 0.3 (abc)	0.076 ± 0.003 (a)	5.3 ± 0.2 (b)	33 ± 2 (bcd)
		**PhT.**	−0.5 ± 0.2 (bcde)	0.034 ± 0.004 (de)	3.8 ± 0.1 (cde)	33 ± 6 (bcd)
		**Recovery**	−0.6 ± 0.2 (cde)	0.064 ± 0.009 (ab)	8 ± 1 (a)	15 ± 1 (f)

Rd, dark respiration rate; Φ CO_2_, quantum yield of CO_2_ uptake; A_max_, maximal rate of net photosynthesis; LCP, light compensation point. These parameters were obtained from the analysis of light response curves, performed at 4°C and 360 ppm of CO_2_ from the Sure 6. Results show mean values ± SE of two separate experiments with 3 replicates each. Different letters represent significant differences between acclimation, treatment and ecotypes *P<*0.05 using three-way ANOVA.

### Total soluble sugar (TSS) and starch in NA and CA ecotypes of *C. quitensis* under cold induced photoinhibition

Both ecotypes store similar amount of TSS and starch in its leaves. However, Antarctic plants present three times higher amount of TSS and twice the amount of starch in roots than Andean plants.

Cold acclimation induced an increment of about 80% of TSS in leaves of both ecotypes (Table 3). The amount of starch increased 66% and 94% in leaves of Andean and Antarctic ecotypes, respectively. In addition, cold acclimation induced 300% increase of the starch content in roots of Andean plants, and 100% in Antarctic plants. Nonetheless, CA Antarctic plants reached 42% greater starch contents than Andean plants.

**Table 3:** **Effect of cold acclimation on TSS (mg g**^**-1**^**DW) and starch (mg g**^**-1**^**DW) changes in leaves and roots of plants of both Andean and Antarctic ecotypes of*C. quitensis*exposed to PhT and recovery period**

			**Leaves**		**Roots**	
**Acclimation**	**Ecotype**	**Treatment**	**TSS**	**Starch**	**TSS**	**Starch**
**Non- acclimated**	**Andean**	**Initial**	53 ± 3(e)	15 ± 2(cde)	21 ± 1(de)	5 ± 2(f))
		**PhT.**	66 ± 3(cd)	13 ± 2(cdef)	13 ± 1(e)	8 ± 2(ef)
		**Recovery**	124 ± 1(a)	11 ± 3(efg)	18 ± 1(de)	5 ± 2(f)
	**Antarctic**	**Initial**	53 ± 2(e)	17 ± 3(cd)	68 ± 7(b)	13 ± 2(bcde)
		**PhT.**	59 ± 1(de)	18 ± 2(c)	27 ± 3(d)	12 ± 3(cde)
		**Recovery**	38 ± 4(f)	13 ± 2(cdef)	27 ± 4(d)	9 ± 2(def)
**Cold- acclimated**	**Andean**	**Initial**	96 ± 1(b)	25 ± 2(b)	58 ± 5(b)	19 ± 2(b)
		**PhT.**	71 ± 6(cd)	7 ± 2(fg)	45 ± 1(c)	15 ± 2(bcd)
		**Recovery**	77 ± 5(c)	10 ± 1(efg)	11 ± 1(e)	13 ± 2(bcde)
	**Antarctic**	**Initial**	99 ± 12(b)	33 ± 3(a)	28 ± 5(d)	27 ± 3(a)
		**PhT.**	59 ± 5(de)	8 ± 2(fg)	29 ± 3(d)	18 ± 2(bc)
		**Recovery**	66 ± 3(cde)	6 ± 2(g)	87 ± 7(a)	14 ± 2(bcde)

Results show mean values ± SE of five different individuals. Different letters represent significant differences between acclimation, treatment and ecotypes *P<*0.05 using three-way ANOVA.

PhT induced an increment of 25% of TSS in leaves of NA Andean plants, while in NA Antarctic plants no changes were observed. NA plants maintained its starch amount in leaves after PhT. TSS decreased in response to PhT and the starch levels were maintained in roots of both NA ecotypes.

Contrasting, under PhT, cold acclimation reduced a 26% and 40% the TSS in Andean and Antarctic plants, and about a 75% the starch levels in the leaves. Under PhT, CA Andean plant maintained the starch amount and reduced a 33% the TSS content, while CA Antarctic plants reduced a 33% its starch level and maintained the TSS.

Under recovery the initial values of TSS were not reestablished, except in NA Andean plants which increased 130%, however, this value was not related with changes in starch amount neither in leaves nor in roots.

## Discussion

### PhT and recovery under light v/s dark conditions

*C. quitensis* from the Andes and Maritime Antarctic are two examples of extreme life under harsh conditions [26,33,34]. While the Andean ecotype is normally subjected to strong seasonal and diurnal temperature oscillations and high PPFD, the Antarctic ecotype grows under permanent low temperatures and occasional high PPFD [26,30-33]. Different environmental adaptation and cold acclimation capacity of these ecotypes has been associated to differences in their resistance to cold-induced photoinhibition [35,38]. Photoinhibition is the reduction of photosynthetic efficiency caused by excess absorbed light. It is the result of a complex interaction between reversible down-regulation of PSII efficiency, damage and recovery capacity of the photosynthetic apparatus [3,39]. Recovery from photoinhibition is strongly limited by low temperatures, mainly by impairment of degradation and “*de novo*” synthesis of D1 [11]. There still lack of clarity in the literature regarding how cold acclimation may help to deal with low temperature-induced photoinhibition [40,41]. Some important questions remain to be addressed, such as whether cold acclimation differentially affects the extent of photoinhibitory damage and/or the recovery capacity after photoinhibition. These two *C. quitensis* ecotypes, which exhibited contrasting freezing tolerance, are good biological models to address the above question.

It is difficult to quantitatively compare the results obtained with *C. quitensis* with those obtained with other species, because different irradiance and temperature have been used in the photoinhibitory treatments. However, under similar or shorter photoinhibitory treatments NA plants of *C. quitensis* exhibited a greater performance than other species. For example, NA plants of *Arabidopsis thaliana*[40], exposed by 2 hours to 600 μmol photons m^-2^ s^-1^, exhibited a reduction of Fv/Fm of about 50%. NA plants of winter wheat [24] experienced a similar decrease in Fv/Fm after 5 hours at 1200 μmol photons m^-2^ s^-1^ and 5°C. In our case, *C. quitensis* only exhibited 25% reduction in Fv/Fm under similar conditions, suggesting a higher resistance to cold-induced photoinhibition in both Antarctic and Andean ecotypes.

Cold acclimation has not changed the extent of Fv/Fm reduction by cold induced photoinhibition in *C. quitensis.* Our results indicate that the recovery process under low-temperature and low light intensity (50 μmol photons m^-2^ s^-1^) was significantly improved by cold acclimation. Moreover, cold acclimation enhanced the recovery process under dark in the cold hardiest ecotype (Antarctic one). It is remarkable, because dark recovery under low temperature imposes significant constraints on photochemical processes and “*de novo”* synthesis of damaged proteins [7]. Full dark recovery after cold induced photoinhibitory treatment in CA plants of *A. thaliana*, takes about 52 hours even at normal growth temperature (22°C) [41]. It is interesting that cold acclimation exhibited a contrasting effect on low light and dark recovery kinetics of both ecotypes at least during the first hours. Cold acclimation promotes a faster restoration of Fv/Fm after PhT in Antarctic than in Andean ecotype. Effective D1 turnover has been indicated as essential for the recovery process from photoinhibition [42]. Low temperature limits the capacity of the repair cycle by decreasing the rate of “*de novo”* protein synthesis and through the inhibition of the proteolytic steps of the D1 repair cycle [43]. Our immunological results showed an 18 kDa D1 degradation product after PhT, suggesting that D1 is being degraded. Although, the process of photodamaged D1 repair is very important during PhT, in *C. quitensis*, it seems it is not a limiting step, because the levels of D1 remained generally constant during PhT and recovery. Only NA Andean plants experienced a decrease in D1, concomitantly with the appearance of 18 kDa D1 degradation product, suggesting a higher susceptibility of this ecotype to cold-induced photoinhibition. This is also consistent with the observed reduction of Cyt *b6/f* after PhT on these plants (Figure 5H). It has been proposed that Cyt *b6/f* level is highly correlated with the electron transport flow, which is mainly limited by plastoquinone re-oxidation at the cytochrome *b6/f* complex [44,45]. Therefore, in Andean plants its decrease could be involved in the decrease of ФPSII and increment of 1-qL during PhT and probably in its poor recovery from photoinhibition.

PSII is generally considered as the major target of photoinhibition, as it is less stable than PSI under strong light treatments. However, several recent evidences showed that PSI can also be affected by photoinhibition. It can occur concomitantly with PSII photoinhibition, especially under chilling conditions and when the linear electron transport chain is unbalanced [46-51]. Cold acclimation induced a better maintenance of PsaA (from RC of PSI) in both ecotypes after PhT. However, during dark recovery PsaA decreased significantly in the Andean ecotype. It seems that PsaA is highly degraded or slowly re-synthesized in the Andean ecotype after PhT. The higher maintenance of PsaA in the Antarctic ecotype could be attributed to the important decrease of antenna proteins Lhca1 and Lhca2. This degradation may cause a decrease in PSI antenna size, thus reducing light absorption efficiency and further decreasing the probability of damage [50]. Antenna complexes are the first target in PSI photoinhibition, similarly to D1 in PSII. It has been proposed that LHCI acts as a safety fuse, being degraded, while the PSI reaction center and its photochemical activity is maintained [51,52].

### Photochemical and non-photochemical processes involved in Fv/Fm restoration

The 25% decreasing in Fv/Fm observed after the PhT evidenced the loss of PSII reaction center activity in both NA and CA plants (Figure 1). However, the data presented clearly demonstrate that cold acclimation increased the recovery capacity by preventing over-reduction of the plastoquinone pool (Figure 2). In CA Antarctic plants this may be due to the maintenance of central proteins from both PS that promote a higher level of ΦPSII (Figure 2) and restricting the photodamage expressed as the slowly relaxing component of NPQ, NPQ_s_ (Figure 3). This response could be the result of acclimation to a high PSII excitation pressure. Therefore, several photosynthetic processes have adapted to this condition increasing their sink capacity at low temperature. Cold-acclimated plants, consistently, presented lower excitation pressure and higher A_max_ and ΦCO_2_ at 4°C compared with NA plants (Table 1, Figure 6). In fact, the ΦCO_2_ at 4°C was 34% higher in CA Antarctic plants than in Andean ones. This was more related with PsaA and Cyt *b6/f* levels considering that D1 level was more stable in both NA and CA plants. These result can be relevant considering the lower light environment where the Antarctic ecotype develops its life cycle. However, both ecotypes presented sustantained photoinhibition, since both decreased ΦCO_2_ and A_max_ after PhT. Similar results have been observed in another Antarctic psychrophile: *Chlamydomonas raudensis* (UWO 241), which also presented an unusually rapid recovery of photosynthetic rate at low temperature [53].

Cold acclimation produced accumulation TSS and starch in leaves and roots of both ecotypes (Table 3). The higher level of starch in leaves and roots was observed in the Antarctic ecotype, reflecting its higher photosynthetic activity with respect to the Andean ecotype. Several lines of evidence suggest that cold-induced sugar accumulation enhances the degree of plant freezing tolerance [54]. The greater amount of TSS and starch in CA plants could be the result of a higher activity of the enzymes involved in photosynthetic carbon metabolism such as Rubisco, F6BP and SPS [55-57].

PhT induced TSS accumulation in NA plants which may be involved in osmotic adjustment of leaves. Contrastingly, PhT in CA plants induced a decrease of TSS and starch in leaves, which could be related with the capability to maintain the functionality of carbon export [58]. It is suggested that this decrease of TSS in leaves could play a role in avoiding the photoinhibition by promoting Calvin cycle enzyme activities [59]. However, this decrease also could be responding to the prolonged time under darkness (12 h), which increases the glycolysis by respiration. On the other hand, the significant reduction of R_d_ during the dark recovery could influence the maintenance of TSS in NA and CA Andean plants, suggesting a differential metabolism activity between these plants and CA Antarctic ones.

Regardless of the effect of PhT on photosynthesis and photochemical processes, after the recovery period, NA plants were also able to reestablish their initial values of A_max_ and ΦCO_2_ in the long term (Table 2, Figure 6) conferring an important role to the fraction of PSII reaction centers that remain active after PhT [60,61]. The surprisingly high level of A_max_ during recovery in CA Antarctic ecotype (70% greater than the initial value in non-photoinhibited plants) are consistent with the increased proportion of open PSII reaction centers and with the maitenance of PsaA and Cyt b6/f even under PhT. This could determine both lineal and cyclic electron transport rate on thylakoids membrane. Cyclic transport induces ATP production by ATP synthase, and lineal electron flow influence the rate of both ATP and NADPH on PSI [62,63], which are necesary for CO_2_ fixation. Cyclic electron transport around PSI has been measured in Antarctic plants under low temperature [64]. The greater cold acclimation-induced stability of thylakoid polypeptides associated to the electron transport chain, which at the end is responsible for ATP and NADPH production, could be the key for the higher reestablishment of photosynthesis upon recovery in Antarctic plants.

NPQ is a mechanism that avoid over reduction of Q_A_[65]. This is a very important protective strategy for both ecotypes under field conditions [35]. In this work, higher values of NPQ and NPQ_f_ were experienced by NA plants in response to chilling (grown at 15°C and measured at 4°C) (Figure 3). It was evident that under PhT, NPQ was unable to keep the Q_A_ pool sufficiently oxidized in neither NA nor CA plants. Lower levels of NPQ after PhT could be explained by the impairment of xanthophyll-cycle deepoxidation at low temperature [66,67], or alternatively because the decrease in electron transport rate, which is necessary to generate ΔpH for Violaxanthin conversion and PsBS reduction [68], wich is well supported by the Cyt *b6/f* decrease in Andean plants. In addition, full NPQ could be limited under darkness and low temperature, because the conversion of Violaxanthin to ABA could be more favored than the conversion to Zeaxanthin [69].

NPQ_s_ has a different behavior with respect to NPQ_f_ (Figure 2). In general, the highest values of NPQ_s_ were measured after PhT. According to Bravo *et al*. [38] the highest values of NPQ in response to 4°C were observed in NA Andean plants (chilling effect). Consistently, in our experiments this ecotype experienced the highest NPQ_s_ levels after PhT (Figure 3). After PhT, NPQ_s_ values were similar to NPQ_f_ values, indicating that NA Andean plants have the highest sensitivity to photoinhibition. This is also consistent with the 30% of D1 reduction level after PhT (Figure 5). The strong correlation between NPQ_s_ and excitation pressures levels (compared to their initial values) suggests that the extent of photoinhibition in *C. quitensis* could be regulated by the redox states of Q_A_ (Figure 4). Cold acclimation did not change the degree of this correlation, indicating that cold acclimation more than reducing the impact of higher excitation pressure, increases the capacity to maintain the electron chain in an oxidized state.

The important down regulation of NPQ observed after PhT and during the recovery may be related with a higher energy partition allocated toward photochemical processes. This is important for the first phase of total Fv/Fm restoration, which is independent of “*de novo”* synthesis protein [25]. The preferential allocation of absorbed energy to photochemistry, together with the D1 level maintenance could help to the accelerated recovery of Antarctic plants under dark conditions.

NPQ, may be suppressed by longer term adjustments, such as the down regulation of some proteins from the antenna complex (Figure 5). CA Antarctic plants strongly reduced the amount of LHCI proteins under PhT and recovery, however not changes on Chl *a*/*b* ratio or LCP were observed. This may be explained, because LHCI does not absorb a great amount of energy, such as LHCII. In contrast, NA plants which decreased both Chl *a* and *b* largely increased their LCP in about 50%. This could be an important strategy to reduce the probability of photoinhibition, decreasing the amount of absorbed energy [14,70].

## Conclusions

Cold acclimation determined full recovery of both ecotypes under darkness and low temperature conditions. Moreover, cold acclimation influenced strongly the velocity of recovery of the Antarctic ecotype under light and darkness. Our results indicate that fast recovery from photoinhibition is related to acquired capacities to maintain electron sinks and repair damage under low temperature of this ecotype upon cold acclimation. The higher performance of the Antarctic over the Andean ecotype was observed in its higher capacity for maintaining D1, PsaA, and Cyt *b6/f* which could contribute to keep lineal electron transport, maintaining lower levels of excitation pressure and NPQ_s_ and a higher capacity for photosynthesis after PhT. These results also suggest that cold-acclimation may stabilize the electron transport chain polypeptides within the thylakoid membrane or maintain their turnover, being an important process for conferring a faster recovery from photoinhibition in the hardiest ecotype. A higher recovery under dark and cold conditions may be a great advantage for the Antarctic ecotype which has a very short growing season. Especially the increased net photosynthesis observed upon cold-acclimation may represent the difference between gaining just enough carbon for vegetative growth or additional carbon to allocate to reproductive organs affecting significantly its fitness.

## Methods

### Plant material and laboratory growth conditions

Antarctic plants of *C. quitensis* were collected on King George Island, Maritime Antarctic (sea level; 62°10’S; 58°29’ W) and transported to the laboratory. The Andean ecotype was collected on the slopes of Cerro La Parva (2.650 m a.s.l.; 33°19’S; 70°17’W). Both ecotypes were reproduced vegetatively in plastic pots, using a soil: peat mixture (3:1 v/v) and maintained at 15°C (near optimal temperature for photosynthesis in both ecotypes) in a growth chamber (Forma Scientific Inc.) with a PPFD of 200 μmol photons m^-2^ s^-1^ and 16 h day length. The light was provided by a mixture of cool-white fluorescent lamps F40CW (General Electric, Charlotte, NC, USA) and white-LED lamps E27/High Power (Ningbo Yinzhou Union Power Lighting Technology, Zhejiang, China) with a maximal peak at 600 nm. One group of each ecotype was cold-acclimated (CA) by transferring it to a growth chamber set at 4°C, for 3 weeks, while the other group was kept at 15°C (non-acclimated, NA). Both CA and NA plants were grown at 200 μmol photons m^-2^ s^-1^, with a 16 h day length and fertilized with 0.12 g L^-1^ Phostrogen per litre of solution (Solaris, Buckinghamshire, UK) once every two weeks.

### Cold- induced photoinhibitory treatment (PhT) and recovery

In order to study the responses under a PhT, NA and CA plants of both ecotypes were transferred to a homemade photoinhibition chamber, consisting of a vertical freezer modified with a glass upper door and, on top of it, a 6 cm thick water filter to prevent radiant heat inside the chamber. Plants were exposed 5 hours to low temperature (4°C) and high PPFD (1.200 μmol photons m^-2^ s^-1^) provided by two 450 watts metal-halide lamps (HQI-TS, Osram, Berlin, Germany) with a emission peak between 500 and 600 nm. The recovery from PhT was performed under darkness at low temperature (4°C). To observe the kinetic of the recovery, Fv/Fm was monitored after 1, 4, 9 and 18 h. Chlorophyll fluorescence, photosynthesis, and proteins were monitored in mature leaves before, immediately after PhT, and at 12 h of the recovery period. This recovery time was chosen because no changes in steady state of Fv/Fm levels were observed after it.

### Chlorophyll fluorescence measurement

Chl fluorescence measurements of *C. quitensis* were performed using a portable fluorimeter (FMS 2, Hansatech Instruments Ltd., Norfolk, UK). Five detached leaves were immobilized with a transparent porous tape as described by Bravo et al. 2007 [38]. Leaves were dark-adapted for 30 min in a humid environment prior to measurements. Measurements were made in a Hansatech LD2/3 leaf chamber (Hansatech, King’s Lynn, UK) at both 15°C and 4°C (to observe the effect of measuring temperature) and at 4°C in plants subjected to PhT. Chamber temperature was controlled by a cooling water bath and monitored continuously during measurements with a thermocouple located just below the leaf lower surface. A moist cloth disk inside the chamber prevents leaf dehydration during measurements. The actinic light used was of 900 μmol photons m^-2^ s^-1^. Fluorescence parameters were calculated as described in Maxwell & Johnson [71] except for qL which was calculated as described in Kramer et al. [72].

NPQ components (NPQ_s_ and NPQ_f_) were calculated from Fm’ dark relaxation kinetics after high light (900 μmoles photons m^-2^ s^-1^) and low temperature (4°C) exposure for a period of 5 hours as previously described by Bravo et al. [37].

### Gas exchange measurements

CO_2_ uptake was measured with an IRGA (CIRAS-2 PP systems, Hitchin, England). Photosynthetic light response curves were recorded every 5 min at 4°C between 0 and 1600 μmol photons m^-2^ s^-1^ at 360 ppm CO_2_. Dark respiration rate (Rd), quantum yield of CO_2_ uptake (ΦCO_2_), maximal rate of net photosynthesis (A_max_), and light compensation point (LCP) were obtained from the analysis of light response curves.

### Western blot analysis of thylakoid proteins

Leaves of *C. quitensis* were ground at 4°C in 20 mM Tricine (pH 7.6) containing 0.4 M sorbitol and 10 mM NaCl, 5 mM EGTA, and 5 mM EDTA with a mortar and pestle. To obtain thylakoids, the leaf brei was filtered through one layer of Miracloth and the filtrate was subsequently centrifuged at 3000 g for 5 min. The pellet was washed in 50 mM Tricine (pH 7.8) 10 mM NaCl, 5 mM MgCl_2_ and centrifuged. Then, thylakoid membranes were resuspended and solubilized in 60 mM Tris (pH 7.8) containing 12% sucrose w/v, 1 mM EDTA, 1% DTT, and 2% SDS. Chlorophyll concentrations were measured according to Arnon [73].

Thylakoid proteins were separated in a SDS-PAGE gel that consisted of a 4% (w/v) stacking gel and a 12% (w/v) solving gel containing 8 M urea. The protein profiles resolved by SDS-PAGE were transferred onto nitrocellulose and immunolabelled with the different antibodies (AgriSera, Vännäs, Sweden). The anti-D1 antibody was raised against the C-termini of the D1 protein; it also allows detection of degradation products of D1. Immuno-detected proteins were developed by enhanced chemiluminescence (ECL) (Pierce, Rockford, USA) on X-ray film (Fuji, Tokyo, Japan). Densitometric measurements of the chemiluminescent bands produced on the X-ray films were quantified with the program ImageJ (NIH, USA). Apparent molecular weights of each protein are showed in the Figure 5.

### Total soluble sugar (TSS) and starch determination

The samples were homogenized and extracted overnight with ethanol (86%). Soluble sugars were measured in the supernadant, while starch was quantificated in the insoluble fraction of ethanolic extractions of soluble sugars, previous acid hydrolysis with perchloric acid 52% v/v. Soluble sugars and starch content was determined spectrophotometrically by the Resorcinol method [74] at 520 nm, using sucrose and starch as respective standards.

### Statistical analysis

Three-way ANOVA (level of significance *P* <0.05) was applied to observe statistically differences due to the following factors: ecotype, acclimation temperature and photoinhibitory-recovery treatment. The response variables were chlorophyll fluorescence parameters, proteins, and light response of gas exchange. Fisher tests were used to identify those means with significant differences. Differences on the kinetic of recovery were determined by one-way ANOVA analysis (*P* <0.05). Statistical analyses were performed using the STATISTICA 6.0 software.

## Abbreviations

ABA: Abscisic acid; A_max_: Maximal rate of net photosynthesis; CA: Cold-acclimated; Chl: Chlorophyll; ΦCO_2_: Quantum yield of CO_2_ uptake; Cyt *b6/f*: Cytochrome *b6/f*; D1: PSII reaction centre protein; ETR: Electron transport rate; F6BP: Fructose-6-Bi-phosphatase; Fv/Fm: Maximum PSII efficiency; ITS: Internal Transcriber Spacers; LT_50_: Lethal temperature at which 50% of plants died; LHCI: Antenna complex from PSI; LHCII: Antenna complex from PSII; Lhca: Light-harvesting protein of the PSI complex; Lhcb: Light-harvesting protein of the PSII complex; LCP: Light compensation point; NA: Non-acclimated; NPQ: Non photochemical quenching; PhT: Cold induced photoinhibitory treatment; PPFD: Photosynthetic photon flux density; PS: Photosystem; 1-qL: Excitation pressure of PSII; ΦPSII: Quantum yield of PSII; Q_A_: Quinone A; RC: Reaction center; ROS: Reactive oxygen species; Rubisco: Ribulose bisphosphate carboxylase/oxigenase; Rd: Dark respiration; SPS: Sucrose phosphate synthase; TSS: Total soluble sugar.

## Authors’ contributions

LBG performed the photoinhibitory assay; take the Chlorophyll *a* fluorescence and photosynthetic measurements and led the writing of the manuscript. CS performed the western-blot. GZ contributed with metabolite assays and discussion. MC propagated and grows both *C. quitensis* ecotypes. LBG and LAB lead the supported projects. LBG, LAB and LJC edited the final manuscript. All authors read and accepted the final manuscript.
